# Folic Acid-Chitosan Conjugated Nanoparticles for Improving Tumor-Targeted Drug Delivery

**DOI:** 10.1155/2013/723158

**Published:** 2013-10-26

**Authors:** Huijuan Song, Chang Su, Wenyu Cui, Bingya Zhu, Liwei Liu, Zhenhua Chen, Liang Zhao

**Affiliations:** ^1^Central Laboratory for Science and Technology, Liaoning Medical University, Jinzhou 121000, China; ^2^College of Veterinary Medicine, Liaoning Medical University, Jinzhou 121000, China; ^3^National Vaccine & Serum Institute, Beijing 100024, China; ^4^College of Pharmacy, Liaoning Medical University, Jinzhou 121000, China

## Abstract

*Objective*. To prepare folic acid-chitosan conjugated nanoparticles (FA-CS NPs) and evaluate their targeting specificity on tumor cells. *Methods*. Chitosan (CS) NPs were prepared by ionic cross linking method, and folic acid (FA) was conjugated with CS NPs by electrostatic interaction. The properties of NPs were investigated, and doxorubicin hydrochloride (Dox) as a model drug was encapsulated for investigating drug release pattern *in vitro*. The cytotoxicity and cellular uptake of FA-CS NPs were also investigated. *Results*. The results reveal that the obtained FA-CS NPs were monodisperse nanoparticles with suitable average size and positive surface charge. Dox was easily loaded into FA-CS NPs, and the release pattern showed a long and biphasic drug release. Noticeable phagocytosis effect was observed in the presence of rhodamine B-labeled FA-CSNPs when incubating with the folate receptor-positive SMMC-7221 cells. *Conclusion*. Compared with the unmodified CS NPs, FA-CS NPs showed much higher cell uptaking ability due to the known folate-receptor mediated endocytosis. FA-CS NPs provide a potential way to enhance the using efficiency of antitumor drug by folate receptor mediated targeting delivery.

## 1. Introduction

Folate receptor (FR) is a kind of receptor existing in malignant tumors, combining with glycosyl phosphatidylinositol (GPI) as a membrane glycoprotein connection [1]. Its expression is highly inhibited in normal tissues, and highly expressed or overexpressed in several human cancers [2]. Expression of folate receptor was established by an *in vitro* model for the investigation of targeted delivery systems [3]. Folic acid (FA) as one of the most popular ligands retains a high affinity for its receptor. Therefore, folic acid and folate conjugates have demonstrated significantly enhanced delivery to FR-positive tumor cells [4–6]. Folic acid or its conjugates combine with folate receptor situated at the surface of cancer cells and are internalized to intracellular compartments to form endosomes [7, 8]. As the conjugation between folate receptor and folate conjugates separates in acid environment (pH = 5.0~5.5), folate receptors return back to the cell surface after dissociation and folate conjugates are degraded by lysosome or released into the cytosol [9, 10]. Chitosan is a linear polysaccharide composed of randomly distributed *β*-(1-4)-linked D-glucosamine (deacetylated unit) and N-acetyl-D-glucosamine (acetylated unit). It is made by treating shrimp and other crustacean shells with the alkali sodium hydroxide. Chitosan is one of the few basic polysaccharides in natural biomaterials, insoluble in water and organic solvents, and soluble in dilute acid solution. Chitosan and its derivatives as natural polymer materials with good biocompatibility, biodegradability, and nontoxic properties have been widely used in the fields of medicine, food, feed, chemical, agriculture, environmental protection, and biotechnology [11–13]. As a new drug delivery and controlled release carrier, nanoparticles have been widely concerned for by researchers in recent years. It can not only enhance the stability of the drug, but can also reach lesion site through the biological barriers, transporting and controlling the release of drug to specific organs and tissues, improving the bioavailability of drugs, and reducing side effects of drugs.

Chitosan nanoparticles have been intensively investigated as a novel drug carrier due to their extensive advantages, such as good biocompatibility, biodegradability, and non-toxic properties [14, 15]. Therefore, chitosan nanoparticles are urgently needed in improving the efficiency of anticancer drug delivery. Herein, in this paper, we aim at preparing folate-chitosan conjugated nanoparticles (FA-CS NPs) for improving tumor-targeted drug delivery. We chose doxorubicin hydrochloride (Dox) as a model drug to investigate the drug encapsulation and release property of FA-CS NPs. The results indicate that the FA-CS NPs have no cell cytotoxicity and can improve the cell uptake of drugs. Our work demonstrates that the obtained FA-CS NPs can be used for potential targeting delivery of anticancer drugs.

## 2. Experimental

### 2.1. Materials

Chitosan (CS, deacetylation degree 80% and molecular weight 400,000) was obtained from Haixin Biological Product Co., Ltd. (China); trimeric sodium phosphate (TPP), acetic acid, folic acid, and rhodamine B were obtained from Sigma Chemicals (St. Louis, US). Doxorubicin hydrochloride was purchased from Beijing Huafeng Lianbo Technology Co., Ltd. (China). All the other chemicals were analytical grade obtained from a variety of vendors. SMMC-7221 cells and MCF-7 cells were obtained from Liaoning Medical University.

### 2.2. Preparation of CS NPs

0.25 g CS was dissolved in 500 mL of acetic acid solution (2%, v/v) by magnetic stirring overnight to prepare 0.5 mg/mL CS solution, and 20 wt% aqueous solution of sodium hydroxide was used to regulate the pH of CS solution to 4.7. CS solution was preheated in 60°C water bath for 10 minutes and then filtrated through 0.45 *μ*m filter to remove the insoluble impurity. TPP reserve liquid (0.5 mg/mL) was prepared and filtrated through 0.45 *μ*m filter. Experiment was performed at controlled ambient temperature 2~8°C, and cold air flow was used to avoid fluctuations in ambient temperature. 10.0 mL of CS solution was added into beaker, and naked nanoparticles were prepared by dropping 3 mL of TPP reserve liquid quickly into the system and continuously stirred for 60 min, until opalescence phenomenon appeared. Prior to the addition of TPP reserve liquid, 10 mL stock solution of Dox at a concentration of 1 mg/mL was added into CS solution to obtain drug-loaded CS NPs. Then, the solution was centrifuged at 16,000 rpm for 15 min, and the supernatant was collected for determining the amount of free Dox. The encapsulation efficiency (EE) of Dox in nanoparticles was determined by measuring the difference between the initially added drug amount and drug in the supernatant calculated using the equation listed below:

(1) EE(%)=((Weight  of  initially  added  drug   −Weight  of  free  drug  in  supernatant)   ×(Weight  of  initially  added  drug)−1)×100.

### 2.3. Preparation and Characterization of FA-CS NPs

5 mg folic acid was dissolved in 10 mL 20 wt% aqueous solution of sodium hydroxide and dropped into 10 mL phosphate buffer suspension (pH 7.4) with 20 mg of CS NPs under oscillation for 30 min. Loading efficiency of FA in CS NPs was determined by measuring the difference between the initially added FA amount and FA in the supernatant. The collected products were washed 3-4 times with deionized water, centrifuged at 16000 rpm for 20 min, and freeze dried to obtain powders. Morphology of particles was observed by using JEM-1200EX (Jeol, Tokyo, Japan) transmission electron microscope (TEM). The mean diameter, zeta potential, and polydispersity index of NPs were determined by Zetasizer (Nano ZS90, Malvern, UK).

### 2.4. Assessment of Drug Release

Accurate weighed 10 mg dried Dox-loaded FA-CS NPs were wrapped in a dialysis bag (spectrum, USA) with 1000 molecular weight and immersed into 100 mL phosphate buffer solution (pH = 7.4) at 37.0 ± 0.5°C under gentle agitation. 5 mL of release medium was withdrawn at each specified time point, and the same volume of fresh buffer solution was added into the release medium to maintain the constant volume. Samples were filtered through 0.45 *μ*m filter and analyzed spectrophotometrically at 484 nm.

### 2.5. Cell Viability Assays

SMMC-7221 cells were applied to investigate cell viability. 150 *μ*L of medium containing SMMC-7221 liver carcinoma cells were added to the 96-well plate at a density of 5 × 10^4^/mL and incubated for 24 h at 37°C under 5% CO_2_. Cells were exposed to suspension of naked FA-CS NPs at different amounts of 0.1, 0.2, 0.3, 0.4, and 0.5 mg/mL. After 24 h, 20 *μ*L MTT with concentration of 5 *μ*g/mL was added into 96-well plate and incubated for 4 h at 37°C. Cultural supernatant was discarded, and 150 *μ*L DMSO was placed in each well and stirred for 30 min. The absorbance of the solution was measured using a microplate reader (Syneray-2, Biotek, USA) at 490 nm.

### 2.6. Uptaking Ability of Different Kinds of NPs in SMMC-7221 Cells and MCF-7 Cells

SMMC-7721 cells and MCF-7 cells were incubated in 6-well plate at 37°C and 5% CO_2_. After 24 h, CS NPs and FA-CS NPs both containing rhodamine B were added into the medium and incubated with cells, respectively. After 6 h, NPs were withdrawn, and wells were washed with PBS two times. The fluorescence intensity was measured using fluorescent microscopy.

## 3. Results

### 3.1. The Preparation and Characteristics of FA-CS NPs

**Figure 1:**
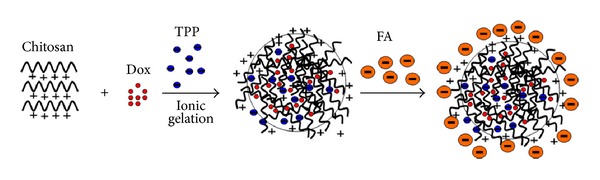
Schematic formation of FA conjugated chitosan Dox-loaded nanoparticles.

**Figure 2:**
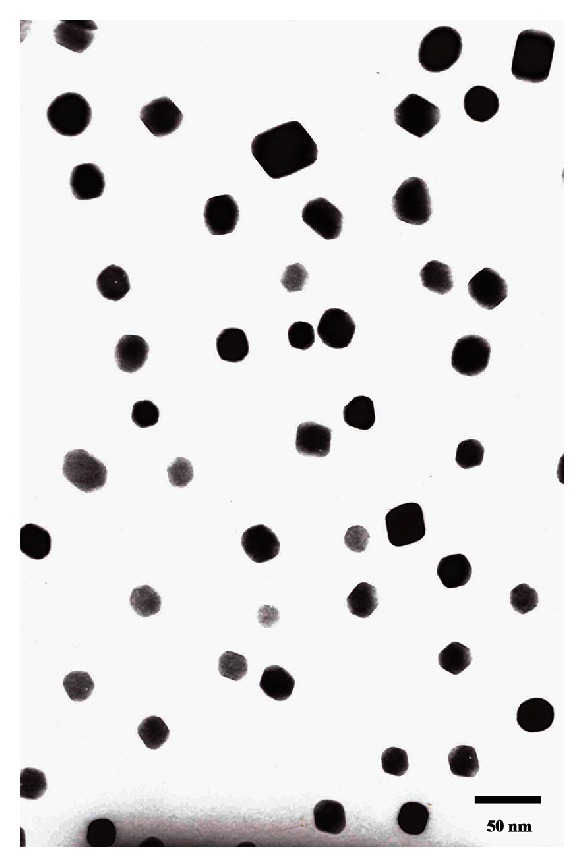
Transmission electron microscopy image of the obtained FA-CS NPs.

CS with protonated amino group aggregated around TPP with negatively charged groups to form particles by electrostatic interactions. Conjugation between CS NPs and FA was due to the fact that cationic amino group of chitosan had strong electrostatic interaction with anionic carboxyl group of FA (Figure 1). Loading efficiency of FA (%) was 30.5 ± 1.2 wt%, and encapsulating efficiency of Dox (%) was 45.4 ± 3.2 wt%. Average diameter, zeta potential, and polydispersity index of NPs were listed in Table 1. Particle morphology of FA-CS NP was analyzed by using TEM images (Figure 2). The results showed that with the conjugation of FA on the surface of CS NPs and Dox-loaded CS NPs, the zeta potentials of both particles were decreased. This might be because FA molecules have modified the surface of the CS particles and the amount of protonated amino group of CS NPs was reduced, leading to the drop of zeta potential. When Dox was encapsulated into CS NPs, its cationic amino groups also enhanced the zeta potential of particles. In term of the polydispersity index (PDI), with the increase of zeta potential of different kinds of NPs, PDI was decreased, and particles with lower PDI showed better stability and monodiepsrsity. The enhancement of zeta potential signified that particles bearing higher charges more easily tended to repel one another instead of aggregation and precipitation, resulting in reducement of PDI and better monodipsersity of NPs. Particle morphology of FA-CS NP was analyzed by using TEM images (Figure 2). The results showed that the obtained FA-CS NPs were monodisperse particles with size that ranged from 12 to 50 nm.

### 3.2. *In Vitro* Drug Release Study

**Figure 3:**
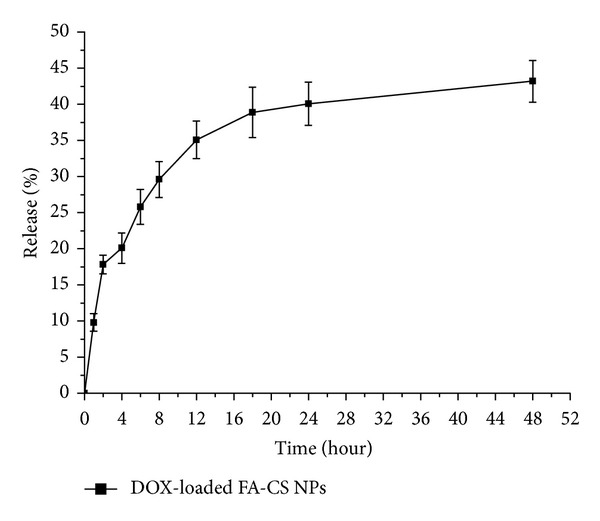
Release profiles of Dox from FA-CS NPs (*n* = 3).

The release pattern of Dox-loaded FA-CS NPs in PBS was divided into initial fast drug release stage and stable release stage (Figure 3). FA-CS NPs showed slight burst release in the first 2 h, implying that drug adsorbed on the surface of nanoparticles entered the medium rapidly. With the medium continuing to diffuse into the interior of NPs, chitosan composed of nanoparticles was slowly degraded; therefore, the drug moved through inside pores in nanoparticles slowly and fell into the medium by diffusion. FA-CS NPs suggested that they had potential as a long-lasting and effective drug delivery system.

### 3.3. Cell Viability Assays

**Figure 4:**
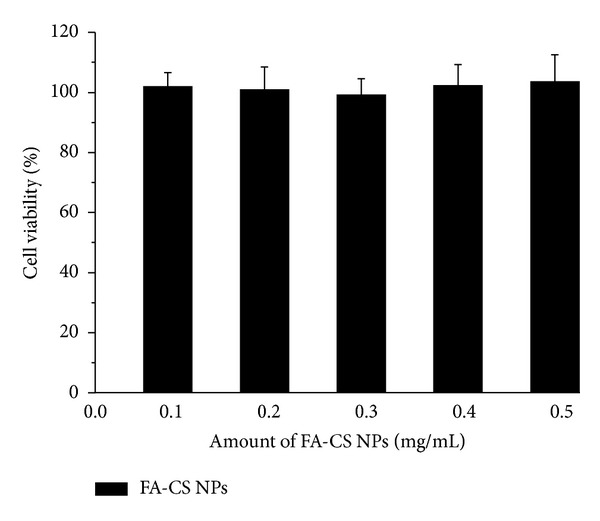
*In vitro* viability of SMMC-7221 cells cultured with different amount of naked FA-CS NPs for 24 h (*n* = 3).

Suspensions containing different amount of naked FA-CS NPs were incubated with SMMC-7721 cells for 24 h, and MTT assay was used to evaluate the cell viability at different amount of NPs. It can be seen from Figure 4 that different amount of naked FA-CS NPs showed no obvious cell inhibition within 24 h.

### 3.4. *In Vitro* Uptake Ability on SMMC-7721 Cells for NPs

**Figure 5:**
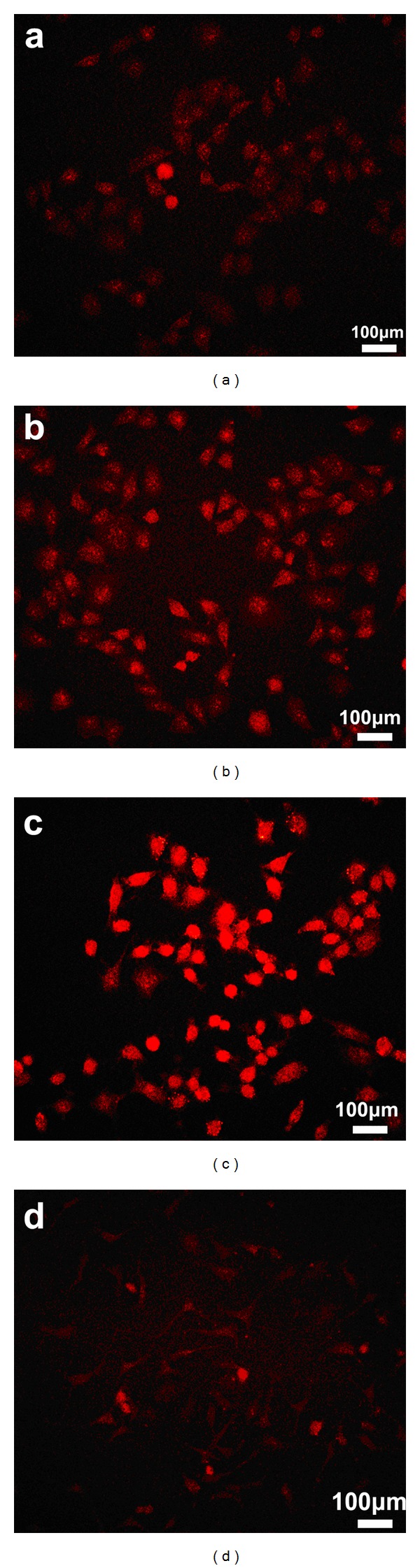
Fluorescent images of SMMC-7721 cells and MCF-7 cells after being incubated for 6 h with different kinds of NPs at the same amount of NPs (0.3 mg/mL), and all of, NPs are labeled by rhodamine B (red). (a) Incubated with CS NPs in SMMC-7721 cells, (b) incubated with unfiltered FA-CS NPs in SMMC-7721 cells, (c) incubated with filtered FA-CS NPs in SMMC-7721 cells, and (d) incubated with filtered FA-CS NPs in MCF-7 cells.

To investigate the effect of FA-mediated endocytosis of NPs, rhodamine B was encapsulated in CS NPs and FA-CS NPs, respectively. Nonencapsulated rhodamine B was removed from suspension containing NPs by dialyzing the mixture against distilled water. Suspension of rhodamine B-labeled CS NPs, filtered rhodamine B-labeled FA-CS NPs, and unfiltered rhodamine B-labeled FA-CS NPs were incubated with SMMC-7721 (a folate receptor-positive cell line) for 6 h (Figure 5). By contrast, filtered rhodamine B-labeled FA-CS NPs were also incubated with MCF-7 cells (a folate receptor-negative cell line) for 6 h to compare the difference of internalizing effects of NPs meditated by FA on cells with different expression of folate receptors. The result showed that more fluorescence can be obviously observed in SMMC-7721 cells incubated with FA modified CS NPs. In contrast, for CS NPs, only low intensity appeared in the interior and peripheral region of the cells. Compared with the low fluorescent intensity of cells incubated with unfiltered FA-CS NPs, more filtered FA-CS NPs tended to gather around the cells and showed intense fluorescent effects in cell interior. In contrast, filtered FA-CS NPs were only slightly internalized by MCF-7 cells after the same duration of incubation (Figure 5(d)). These findings were in good agreement with the observation via fluorescence microscopy.

## 4. Discussion

In order to achieve coupling of FA on nanoparticles, a large amount of organic reagents and toxic coupling reagent were commonly used, thus leaving more residual coupling reagent and resulting in cell toxicity. However, conjugation between CS NPs and FA was due to the fact that cationic amino group of chitosan had strong electrostatic interaction with anionic carboxyl group of FA. Therefore, the process of obtaining FA-CS NPs in our work avoided the addition of toxic organic solvents.

The obvious differences on cell uptaking ability of different kinds of nanoparticles may be due to the fact that phagocytosis of nanoparticles may be relative to folate-receptor-mediated interaction. In term of SMMC-7721 cells in which folate receptors were overexpressed, FA modified in CS NPs was combined with folate receptor through the ligand receptor interaction, and cells tended to internalize nanoparticles more easily. Free FA existing in the suspension of unfiltered FA-CS NPs was competing with FA conjugated with CS NPs to bind the folate receptor on the surface of cells, resulting in the saturation of receptors and reduction of endocytosis of NPs. As there were no folate receptors on MCF-7 cells, the conjugation of FA on CS NPs took no obvious improving effects on the internalization of NPs.

## 5. Conclusion

In summary, monodisperse FA-CS NPs with the average size of 38 ± 2 nm and surface potential of 13.6 ± 4.8 mV were facile prepared. Dox was easily loaded to FA-CS NPs with the encapsulation efficiency of 45.4 ± 3.2 wt%. The release data showed that Dox loaded FA-CS NPs had a long and biphasic release behavior. Compared to the lower uptake of CS NPs in tumor cells, more FA-CS NPs with no obvious cytotoxicity within 24 h can be phagocytosed by folate receptor-positive tumor cells. Our results indicate that FA-CS NPs can be a promising tumor-targeting carrier candidate.

## Figures and Tables

**Table 1:** Key parameters of NPs.

Parameters	FA-CS NPs	DOX-loaded FA-CS NPs	CS NPs	DOX-loaded CS NPs
Average diameter (nm)	38 ± 2	43 ± 5	29 ± 3	31 ± 3
Zeta potential (mV)	13.6 ± 4.8	19.2 ± 6.9	25.5 ± 5.9	30.4 ± 8.7
Polydispersity index	0.110	0.084	0.064	0.052
